# Supplementary material for: Sonographic Assessment of Complex Ultrasound Morphology Adnexal Tumors in Pregnant Women with the Use of IOTA Simple Rules Risk and ADNEX Scoring Systems
Source: Diagnostics (Basel). 2021 Feb 28;11(3):414. doi: 10.3390/diagnostics11030414 (PMC7997447; doi:10.3390/diagnostics11030414)
Supplement: Supplementary file 1 [file diagnostics-11-00414-s001.zip › diagnostics-1088466/diagnostics-1088466-supplementary file S2.docx]

Supplementary material 2.

Sonographic assessment of complex adnexal masses in pregnant women with the use of IOTA Simple Rules Risk and ADNEX scoring systems.

A.Czekierdowski, N.Stachowicz, Smoleń A, T.Kluz, T.Łoziński, Miturski A, Kraczkowski J.

Overview of prediction models

| **Model** | **Publication year** | **Variables in model** | **Type of model** |
| --- | --- | --- | --- |
| **ROMA** | 2009 | CA125 and HE4, menopausal status | Logistic regression model providing a risk of malignancy |
|  |  |  |  |
| **SRRisk** | 2016 | Unilocular cyst, largest diameter of largest solid component <7 mm, acoustic shadows, smooth multilocular cyst with largest diameter <100 mm, no intra-tumoral blood flow at color or power Doppler, irregular solid tumor, ascites, at least four papillary projections, irregular multilocular solid tumor with largest diameter ≥100 mm, very strong intra-tumoral blood flow at color or power Doppler, type of center (oncology or non-oncology center). | Logistic regression model providing a risk of malignancy. |
| **ADNEX** | 2014 | Patient´s age, type of center (oncology or non-oncology center), serum CA-125 (not mandatory), maximum diameter of lesion, maximum diameter of largest solid component, more than 10 cyst locules, number of papillary projections, acoustic shadows, ascites | Logistic regression model providing the likelihood of a benign tumor and four malignant tumor subtypes (borderline, stage I primary malignancy, stage II–IV primary malignancy, metastasis in the ovary from another primary malignancy); the risk of malignancy is the sum of the risks of each malignant subtype. |

ROMA= Risk of Malignancy Algorithm, SRRisk= Simple Rules Risk model; ADNEX= Assessment of Different NEoplasias in the adnexa

**1.Risk of Ovarian Malignancy Algorithm (ROMA)** was proposed in 2009 by Moore et al.who combined two tumor marker levels: HE4 and CA125 levels and developed logistic regression model that calculates ovarian cancer risk according to the menopausal status, defined by lack of menstruation or clinical signs of menopause for 6 months.

Pre−menopausal Predictive Index(PI)=−12.0+2.38×LN(HE4)+0.0626×LN(CA125)

Post−menopausal Predictive Index(PI)=−8.09+1.04×LN(HE4)+0.732×LN(CA125)

Predicted Probability(PP)=exp(PI)/[1+exp(PI)]×100

Moore RG, McMeekin DS, Brown AK, et al. A novel multiple marker bioassay utilizing HE4 and CA125 for the prediction of ovarian cancer in patients with a pelvic mass. Gynecol Oncol. 2009;112:40–46. doi: 10.1016/j.ygyno.2008.08.031.

 According to the immunological assay for measuring CA125 and HE4, the cut off levels can differ to classify patients into either a low or an high risk group. With the Roche Diagnostics Laboratory’s ECLIA method, the cut off level to classify patients in a high risk group was 11.4% for pre-menopausal patients, and 29.9% for menopause patients, whereas with Abbott Diagnostics Laboratory’s CMIA method, the cut off levels were respectively 7.4% and 25.3%

**2.Simple Rules risk model (SRRisk)**

SRRisk is a logistic regression model that was published in 2016. It is based on data from 4848 patients recruited at 22 centres in Belgium, Sweden, Italy, Czech Republic, Poland, Spain, United Kingdom, China, and Canada (Timmerman et al 2016). The model is based on ten ultrasound features and the type of center at which the patient is examined. Five ultrasound features are indicative of a benign tumor: unilocular cyst (‘B1’), presence of solid areas smaller than 7mm in largest diameter (‘B2’), presence of acoustic shadows (‘B3’), smooth multilocular tumor with largest diameter less than 100mm (‘B4’), no intra-tumoral blood flow (‘color score’ 1, ‘B5’). Five ultrasound features are indicative of a malignant tumor: irregular solid tumor (‘M1’), presence of ascites (‘M2’), presence of at least four papillary structures (‘M3’), irregular multilocular-solid tumor with largest diameter ≥100 mm (‘M4’), and very strong intra-tumoral blood flow (‘color score’ 4; ‘M5’). The ultrasound terminology and the measurement technique described in the IOTA ‘terms and definitions’ statement are used (Timmerman et al 2000). Type of center is a binary variable indicating whether the patient was examined at an oncology center or not (1 vs 0; ‘oc’). Oncology center is defined as a tertiary referral center with a specific gynecological oncology unit. Oncology centers usually have a higher prevalence of malignant tumors, and this should be acknowledged in the risk estimate.

SRRisk is based on a logistic regression model with a random intercept for center. The final formula sets the random intercepts to zero, and hence uses only the fixed intercept. The formula is

$$risk=\frac{exp\left( z \right)}{1+exp\left( z \right)}$$

where:

$$z=-0.9713-3.4059*b1-2.2520*b2-1.6633*b3-2.7469*b4-1.8624*b5$$

$+ 2.1933*m1+2.6540*m2+1.5308*m3+0.9806*m4+1.5476*m5+0.9186*oc$.

Note that four decimals are used for every coefficient, whereas the SRRisk article mentions only two. This has no impact on the results.

**3.The Assessment of Different NEoplasias in the adneXa (ADNEX) model**

The ADNEX model is a multinomial logistic regression model that was published in 2014. It is based on data from 5909 patients recruited at 25 centers in Belgium, Sweden, Italy, Czech Republic, Poland, France, Spain, United Kingdom, China, and Canada (Van Calster et al 2014). ADNEX estimates the risk of five types of tumor: benign, borderline, stage I primary ovarian malignancy, stage II-IV primary ovarian malignancy, and metastasis in the ovary from another primary malignancy. The model is based on nine clinical and ultrasound variables: age of the patient (in years), serum CA125 (U/mL) (optional), maximum diameter of the lesion (in mm; ‘mdl’), the proportion of solid tissue calculated as the maximum diameter of the largest solid component (in mm) divided by the maximum diameter of the lesion (value between 0 and 1; ‘pst’), presence of more than 10 cyst locules (1 versus 0; ‘tcl’), the number of papillary structures (0, 1, 2, 3, 4, with 4 indicating more than three; ‘nps’), presence of acoustic shadows (1 versus 0; ‘sha’), the presence of ascites (1 versus 0; ‘asc’), and examination at an oncology center (1 versus 0; ‘oc’). The ultrasound terminology and the measurement technique described in the IOTA ‘terms and definitions’ statement (Timmerman et al 2000) are used.

ADNEX is based on a multinomial logistic regression model with random intercepts for center. The final formula sets the random intercepts to zero, and hence uses only the fixed intercepts. A version of ADNEX without CA125 was also developed, because CA125 is not measured routinely in every center. The formula of ADNEX with CA125 is:

$${risk}_{benign}=\frac{1}{1+exp\left( z_{1} \right)+exp\left( z_{2} \right)+exp\left( z_{3} \right)+exp\left( z_{4} \right)}$$

$${risk}_{borderline}=\frac{exp\left( z_{1} \right)}{1+exp\left( z_{1} \right)+exp\left( z_{2} \right)+exp\left( z_{3} \right)+exp\left( z_{4} \right)}$$

$${risk}_{stage I cancer}=\frac{exp\left( z_{2} \right)}{1+exp\left( z_{1} \right)+exp\left( z_{2} \right)+exp\left( z_{3} \right)+exp\left( z_{4} \right)}$$

$${risk}_{stage II-IV cancer}=\frac{exp\left( z_{3} \right)}{1+exp\left( z_{1} \right)+exp\left( z_{2} \right)+exp\left( z_{3} \right)+exp\left( z_{4} \right)}$$

$${risk}_{secondary metastasis}=\frac{exp\left( z_{4} \right)}{1+exp\left( z_{1} \right)+exp\left( z_{2} \right)+exp\left( z_{3} \right)+exp\left( z_{4} \right)}$$

where

$$z_{1}=-7.577663+0.004506*age+0.111642*log2\left( ca125 \right)+0.372046*log2\left( mdl \right)$$

$$+ 6.967853*pst-5.65588*{pst}^{2}+1.375079*tcl+0.604238*nps$$

$$- 2.04157*sha+0.971061*asc+0.953043*onc$$

$$z_{2}=-12.276041+0.01726*age+0.197249*log2\left( ca125 \right)+0.87353*log2\left( mdl \right)$$

$$+ 9.583053*pst-5.83319*{pst}^{2}+0.791873*tcl+0.400369*nps$$

$$- 1.87763*sha+0.452731*asc+0.452484*onc$$

$$z_{3}=-14.91583+0.051239*age+0.765456*log2\left( ca125 \right)+0.430477*log2\left( mdl \right)$$

$$+ 10.37696*pst-5.70975*{pst}^{2}+0.273692*tcl+0.389874*nps$$

$$- 2.35516*sha+1.348408*asc+0.459021*onc$$

$$z_{4}=-11.909267+0.033601*age+0.276166*log2\left( ca125 \right)+0.449025*log2\left( mdl \right)$$

$$+ 6.644939*pst-2.3033*{pst}^{2}+0.89998*tcl+0.215645*nps$$

$- 2.49845*sha+1.636407*asc+0.808887*onc$.

For ADNEX without CA125, use

$$z_{1}=-7.412534+0.003489*age+0.430701*log2\left( mdl \right)$$

$$+ 7.117925*pst-5.74135*{pst}^{2}+1.343699*tcl+0.607211*nps$$

$$- 2.11885*sha+1.167767*asc+0.983227*onc$$

$$z_{2}=-12.201607+0.017607*age+0.98728*log2\left( mdl \right)$$

$$+ 10.07145*pst-6.17742*{pst}^{2}+0.763081*tcl+0.410449*nps$$

$$- 1.98073*sha+0.77054*asc+0.543677*onc$$
